# Genomic analysis of *Sweet potato feathery mottle virus* from East Africa

**DOI:** 10.1016/j.pmpp.2020.101473

**Published:** 2020-04

**Authors:** Godfrey Wokorach, Geoffrey Otim, Joyce Njuguna, Hilary Edema, Vincent Njung'e, Eunice M. Machuka, Nasser Yao, Francesca Stomeo, Richard Echodu

**Affiliations:** a Biosciences Research Laboratory, Gulu University, P.O. Box 166, Gulu, Uganda; b Faculty of Agriculture, Gulu University, P.O. Box 166, Gulu, Uganda; c Biosciences Eastern and Central Africa, International Livestock Research Institute (BecA-ILRI) Hub, P.O. Box 30709, Nairobi, 00100, Kenya; d Department of Biology, Faculty of Science, Gulu University, P.O. Box 166, Gulu, Uganda

**Keywords:** Sweet potato, Potyvirus, East Africa, Recombination, SPFMV, RC, Russet crack, EA, East Africa, O, ordinary, SPFMV, Sweet potato feathery mottle virus

## Abstract

*Sweet potato feathery mottle virus* is a potyvirus that infect sweet potato. The genome of the virus was analysed to understand genetic diversity, evolution and gene flow. Motifs, nucleotide identity and a phylogenetic tree were used to determine phylogroup of the isolates. Gene flow and genetic diversity were tested using DnaSP v.5. Codons evolution were tested using three methods embedded in Datamonkey. The results indicate occurrence of an isolate of phylogroup B within East Africa. Low genetic differentiation was observed between isolates from Kenya and Uganda indicating evidence of gene flow between the two countries. Four genes were found to have positively selected codons bordering or occurring within functional motifs. A motif within *P1* gene evolved differently between phylogroup A and B. The evidence of gene flow indicates frequent exchange of the virus between the two countries and *P1* gene motif provide a possible marker that can be used for mapping the distribution of the phylogroups.

## Highlights

• Phylogenetic tree indicates the present of phylogroup B in east Africa for the first time.
• Evidence of gene flow was detected between isolates from Kenya and Uganda.
• Recombination events were common among isolates of phylogroup A than phylogroup B.
• Nucleotide repeat motif within P1 gene evolved differently between phylogroup A and phylogroup B.

## 1 Introduction

Sweet potato feathery mottle virus (SPFMV; species *Sweet potato feathery mottle virus*, genus *Potyvirus*), belongs to the largest and most important family of plant viruses, *Potyviridae* [1,2]. SPFMV is the most significant potyvirus of sweet potato (*Ipomoea batatas* L.), occurring worldwide [3,4]. In East Africa, it occurs in all agro ecological zones where sweet potato is grown, especially around the Lake Victoria Basin [5,6]. According to older classification texts, SPFMV can be grouped into strains O, EA, and RC [7]. Plant virus classification based on geographical location and biological property such as the naming of SPFMV strains (i.e. O, EA, and RC) was considered misleading [8]. More recent examination of SPFMV groups put forward a new classification proposal and grouped the isolates into phylogroups A and B [9]. Phylogroup A comprised strain O and strain EA isolates. Phylogroup B consisted of strain RC isolates. Strain EA isolates were first detected in East Africa and Madagascar where they appeared to be localized, forming the major SPFMV virus group [7]. Later studies detected the occurrence of strain EA in Peru and Dili, East Timor [9,10]. Strain O and RC had a wider geographical distribution and occurrence but with limited occurrence within the East African bloc [11]. Occurrence of phylogroup B (strain RC) in East Africa had not been reported.

Sweet potato plants infected with SPFMV only usually present mild or no symptoms [4]. However, severe symptoms become evident when SPFMV co-infects with *Sweet potato chlorotic stunt virus* (genus *Crinivirus*, family *Closteroviridae*), resulting in the disease syndrome known as sweet potato virus disease (SPVD). This is the most devastating viral disease reported in sweet potato and causes yield losses of up to 98% in some cultivars [5]. Both SPFMV and *Sweet potato chlorotic stunt virus* (SPCSV) have higher prevalence and distribution across East Africa than any of the sweet potato viruses reported to infect sweet potato in East Africa [5,12,13].

East Africa has been described as a centre of diversity and evolution for major plant viruses. High genetic variability among SPFMV isolates from East Africa have already been reported [11]. The high genetic variability among virus isolates present difficulties with detection methods and consequently present challenges with their control strategies [3]. The severity of SPFMV infection on the different host plants is isolate/variant specific [14] and is influenced by the diversity and variability among SPFMV. Knowledge on the presence and distribution of different variants and phylogroup help to determine how different variant group influence the manifestation of symptoms and yield loses in sweet potato in East Africa. Host resistance is a strategy to manage burden caused by sweet potato viruses. However, host resistance mechanisms tend to favour native strain/phylogroup of the virus; thus, the introduction of a new SPFMV phylogroup may have serious consequences on sweet potato production in East Africa. Understanding the different SPFMV phylogroup in East Africa, their occurrence, distribution, diversity and evolution pattern can provide important information to guide current and future management strategies. The aim of this study was to understand the different SPFMV phylogroups, their genetic diversity, and evolution in East Africa.

## **2** Materials and methods

### 2.1 Sampling

Sweet potato fields were surveyed in four East African countries – Kenya, Rwanda, Tanzania and Uganda – between June 2015 and November 2016. Fields were selected by systematic random sampling at an interval of 2 km along roads. In each field, vines from symptomatic plants were selected along an X-patterned transect. The vines were grown inside an insect-proof screen house at Gulu University, Uganda. Leaves from symptomatic vines were collected, immediately preserved in liquid nitrogen and transported to the Biosciences eastern and central Africa-International Livestock Research Institute (BecA-ILRI) Hub for RNA isolation and sequencing. In total, leaf samples from 96 sweet potato plants from the four countries were used for library preparation and sequencing.

### 2.2 RNA extraction

Total RNA was extracted using the ZR Plant RNA MiniPrep kit (Zymo Research, Irvine, CA, USA) following the manufacturer's protocol. The quality of the extracted RNA was evaluated by electrophoresing on 1% agarose gel. The concentration was determined with a Qubit 2.0 fluorometer (Life Technologies, Carlsbad, CA, USA) as described in the manufacturer's protocol.

### 2.3 Library preparation

RNA library construction was performed using the Illumina TruSeq RNA sample-preparation kit (Illumina, San Diego, CA, USA). Briefly, mRNA was purified from 0.4 μg of total RNA using Illumina RNA purification beads. Purified mRNA was subjected to first-strand cDNA synthesis using SuperScript™ II reverse transcriptase (ThermoFisher Scientific, Waltham, MA, USA) and first-strand master mix. Subsequently second strand synthesis followed using second-strand master mix, both provided with the kit. This was followed by end-repair and subsequent adenylation of the 3′ end to prevent fragment ligation before ligation of Illumina adaptors. Finally, the libraries underwent 15 cycles of PCR and quality were confirmed using an Agilent 2200 tape station (Agilent Technologies Inc., Santa Clara, CA, USA). The average peak size for the 96 libraries prepared was 298.99 bp.

### 2.4 Library normalisation and sequencing

Sequencing was done with a MiSeq Reagent Kit v.3 (600 cycle) using the manufacturer's reagents. All libraries were diluted to 4 nM and pooled together. Five microliters of 0.2 N sodium hydroxide was added to denature 5 μl of the pooled libraries. Denatured libraries were diluted to a final concentration of 7 pM with HT1 buffer, spiked with 20 pM of 1% PhiX control and a volume of 600 μl loaded onto the sequencing cartridge. The libraries were paired end sequenced (2 × 151) using the Illumina MiSeq v.2 located at the BecA-ILRI Hub.

### 2.5 Sequence data analysis pipeline

The quality of the generated reads was checked using FastQC Babraham Bioinformatics, https://www.bioinformatics.babraham.ac.uk/projects/fastqc/. Low-quality reads, Illumina adapter indices and PCR primers were removed using Trimmomatic v.0.33 [15]. The plant host reads were removed by mapping to the genome of *Ipomoea trifida* using Bowtie 2.3.3 [16]. Reads that did not mapped to the genome of *Ipomoea trifida* after indexing were assembled into scaffolds using SPAdes 3.10.1 [17]. Using BLASTn [18], we checked the assembled *de novo* scaffolds against the NCBI plant virus database. The BLASTn output was visualized graphically using Krona v.2.4 [19], which summarized the viruses present in each sample. The assembled scaffolds of SPFMV were uploaded into CLC Genomics Workbench v.8 (https://www.qiagenbioinformatics.com) for annotation against a reference sequence AB465608.1 [20] obtained from GenBank. Sequences KF386013 and KF386014 isolated from Argentina [35], AB509454, AB439206 and D86371 isolated from Japan [20,36], KP729265 isolated from Uganda [37], FJ155666 isolated from Peru [38], MF572055 and MF572056 isolated from east Timor, MF572047 isolated from Australia [9], and KP115608 isolated from Korea [39] were used for comparison with east Africa isolates. Phylogenetic tree was done using MEGA7 [21]. Best DNA model for phylogenetic tree construction was calculated in MEGA7. General Time Reversible plus discrete Gamma distribution (+G) and evolutionarily invariable (+I) was the best model that described the substitution pattern for the phylogenetic tree. Bayesian information criterion score, Akaike information criterion score, and maximum likelihood score for the selected model were 120639.7461, 119902.5581, and −59882.2641 respectively. The phylogenetic tree was done with a setting of 1000 bootstrap replicates. Pair wise nucleotide identity were calculated in CLC Genomics Workbench v.8.

### 2.6 Nucleotide diversity and genetic differentiation

Nucleotide diversity, number of segregating sites, average number of nucleotide differences, and total number of mutations between sub-populations were analysed in DnaSP v. 5 [22]. Genetic differentiation on full length of polyprotein encoding nucleotide sequence was analysed for the different sub-populations. Fixation indices *Fst* was used to determine genetic differentiation and gene flow between different sub-populations. Neutrality test based on Tajima's and Fu & Li's parameters were done, firstly for all the isolates and subsequently on different sub-populations.

### 2.7 Episodic positive selection within genes of SPFMV

Detection of sites under positive diversifying selection or purifying selection was done in Datamonkey (Viral evolution group, University of California, San Diego, USA) online server. IFEL (internal branches fixed-effects likelihood), REL (random effects likelihood) and MEME (mixed effects model of evolution) [23,24] were used to identify sites under positive selection. The default threshold p-value was used to confer site under positive selection.

### 2.8 Recombination analysis

Recombination events on SPFMV genome were predicted using RDP v.4.95 [25]. A full exploratory recombination scan on the aligned SPFMV genome was performed with six methods (RDP, chimaera, BootScan, GENECONV, MaxChi, and SiScan). Settings for both BootScan and SiScan were as follows: BootScan window size = 200 and step size = 20. Additional BootScan settings were the following: bootstrap replicates = 100, random number seed = 3 and cut off percentage = 100. Parameters used in SiScan were a p-value permutation of 1000 and a scan permutation number of 10. Window size for RDP was set at 30. Variable sites per window was 70 for MaxChi and 60 for Chimaera. Recombination events predicted by the six algorithms with p-value (<0.05) were considered valid.

## 3 Results

### 3.1 Genome properties of SPFMV

A total of 45,426,299 reads were obtained but the number was reduced to 33,837,419 after quality trimming. Sixteen complete SPFMV genomes were obtained from *de novo* assembly. The genome sizes range was 10,803–10,891 nucleotides, excluding the poly (A) tail. The polyprotein length for the isolates was 10,557 nucleotides − except for isolate P33 which had a polyprotein length of 10,482 nucleotides (Table S1). Translation of the polyprotein produced 3518 amino acids for all the isolates − except P33 which had 3493 amino acids (Table S1). In all isolates, the 5′ UTR region was shorter (length range 90–116 nucleotides) compared with the 3′ UTR region (length range 207–221 nucleotides).

### 3.2 Phylogenetic analysis

**Fig. 1:**
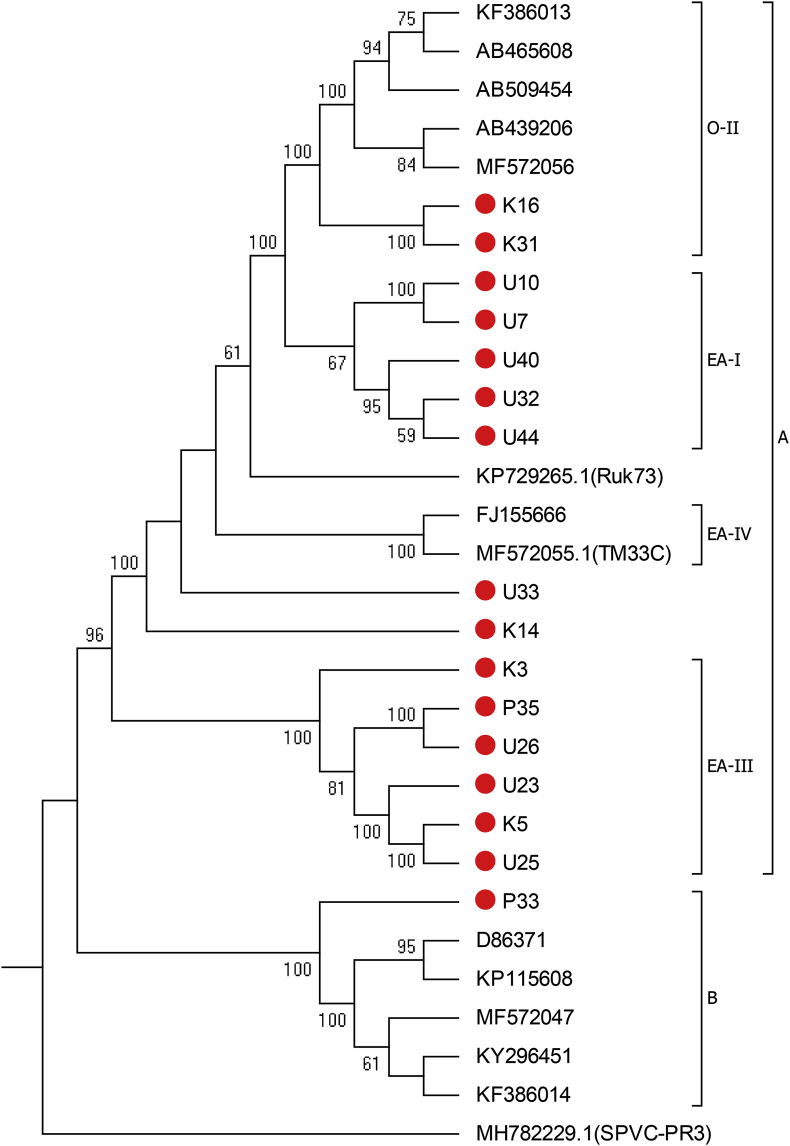
Phylogenetic tree of polyprotein encoding nucleotide sequence. Reference isolate MH782229.1 of sweet potato virus C was used as out group. Red colour indicate isolates from this study. (For interpretation of the references to colour in this figure legend, the reader is referred to the Web version of this article.)

The maximum-likelihood phylogenetic tree done from polyprotein encoding nucleotide sequences clustered the isolates into two major phylogroup A and B. Phylogroup A had four sub-clusters: EA-I, EA-III, and EA-IV (strain EA) and O-II (strain O). Isolates K16 and K31 clustered with reference isolates of O-II. Isolate P33 clustered with reference isolates of phylogroup B (strain RC). EA-III comprised isolates from Uganda (U26, U23, and U25), Kenya (K5 and K3) and Rwanda (P35) while EA-I had isolates from Uganda only (Fig. 1). EA-IV consisted of two strain EA isolates (FJ155666 and MF572055) from Peru and Dili, East Timor. Isolates U33, K14, and KP729265.1 (Ruk 73) did not cluster with any subgroup.

### 3.3 Polyprotein encoding nucleotide sequence identity with reference sequences

Nucleotide identity test against known reference isolates, indicate that Kenya isolates K31 and K16 shared a nucleotide identity of 96.33–96.51% with isolates AB465608.1 and KF386013.1 from subgroup O-II and 92.13–92.20% identity with FJ155666.1 of EA-IV. Isolates K31 and K16 shared 87.09–87.24% identity with D86371.1 and KF386014.1 of phylogroup B (strain RC). Isolate P33 shared a 92.28–92.45% nucleotide identity with phylogroup B isolates (D86371.1 and KF386014.1), 86.79–86.82% with AB465608.1 and KF386013.1 of O-II subgroup, and 86.68% with FJ155666.1. Phylogroup A had 2,067 nucleotides within the *P1* gene, while isolate of phylogroup B had only 1,992 nucleotides. Phylogroup B including P33 isolate had a deletion of 75 nucleotides corresponding to positions 875–949 within the *P1* gene of phylogroup A. Translation of the *P1* gene produced a protein of 689 amino acids in phylogroup A and 664 amino acids in phylogroup B. EA-IV had 724 amino acids within *P1 gene*. A repeat of six Q amino acids was common among all isolates at positions 32–37 except for isolates U32 and U40, which had amino acid KE at positions 34 and 35. Amino acid block KRTV at positions 623–628 and LEPV positions 269–272 were conserved within *P1 gene* of phylogroup B. Tripeptide APT at positions 312–314, EPT at positions 327–329, dipeptide TM at 629–630 and LR at 663–664 were unique and conserved within *P1 gene* of phylogroup B. Similarly, dipeptides LR at positions 23–24, SR at positions 79–80 and SE at positions 87–88 within *HC-Pro* gene were unique to isolates of phylogroup B.

### 3.4 Nucleotide diversity among sub-populations of SPFMV

Nucleotide diversity for 29 polyprotein encoding nucleotide sequences was 0.087; the value higher than diversity measures within the different sub-populations (Table 1). Nucleotide diversity were high for EA isolates from East Africa compared with other subgroups. Uganda isolates (EA group) had diversity of 0.069 and Kenya isolates (EA group) had diversity of 0.057. An overall genetic variability of 0.034 for phylo-group B. Similarly, O-II had a diversity score of 0.031. Diversity of O-II of East Africa origin (K16 and K31) alone was 0.022; this is similar to that obtained for O-II of non-East African origin, i.e. 0.024. O-II and EA-I had the lowest average number of nucleotide differences (562.800), followed by the differences between strain EA-I and EA-IV (659.500). Phylogroup B had the highest nucleotide difference compared to other subgroups ranging between 1485.889–1200.083.

**Table 1:** Population statistics based on variation within polyprotein encoding nucleotide sequence sequences.

	Sample size	Nucleotide diversity	number ofsegregating sites	Average number of nucleotide differences	Total number of mutations ᶿ
All isolates	29	0.087	3459	909.341	3993
EA-I	5	0.038	950	400.533	968
EA-III	6	0.039	1159	412.424	1188
O-II	7	0.031	948	322.725	973
B	6	0.034	1099	357.924	1119
Uganda isolates (Strain EA)	10	0.069	2166	729.768	2319
Kenya Isolates (Strain EA)	3	0.057	1122	604.533	1145
O-II East Africa origin (K16 and K31)	2	0.022	343	228.666	343
O-II (non-East Africa origin)	5	0.024	604	246.577	613

### 3.5 Genetic differentiation between sub-population

Isolates from Kenya and Uganda provided evidence of gene flow: *Fst* = 0.162 for polyprotein encoding nucleotide sequence and *Fst* = 0.014 for coat protein (Table 2). Phylogroup B had *Fst* = 0.727 with O-II, *Fs*t = 0.707 with EA-I, and *Fst* = 0.7746 with EA-III. Isolates of O-II and EA-I had genetic differentiation of *Fst* = 0.367. K16 and K31 had high genetic differentiation with O-II of non-East Africa origin (Table 2). EA-IV show high genetic differentiation with both EA-I and EA-III for polyprotein length. *Fst* = 0.154 score was for coat protein of EA-IV and EA-III (Table 2).

**Table 2:** Genetic differentiation among sub-population based on polyprotein and coat protein.

Group 1	Group 2	*Fst* for Polyprotein	*Fst* for Coat protein
O-II	Phylogroup B	0.727	0.738
O-II	EA-I	0.367	0.652
O-II	EA-III	0.699	0.607
Phylogroup B	EA-I	0.708	0.595
Phylogroup B	EA-III	0.745	0.496
EA-I	EA-III	0.638	0.245
O-II East African origin (K16 and K31)	O-II (non-East Africa origin)	0.441	0.488
Uganda isolates (strain EA)	Kenya isolates (strain EA)	**0.162**	**0.014**
EA-IV	EA-I	0.532	0.409
EA-IV	EA-III	0.691	**0.154**

Bold indicate low genetic differentiation between the sub-population.

### 3.6 Neutrality test among sub-population

Neutrality test performed on all isolates indicate positive Tajima's D value 0.223 though this was not significant. Fu & Li's neutrality test for all isolates were positive and significance D* = 2.389, *P < 0.02* and F* = 1.827, *P < 0.05* (Table 3). Neutrality testing was further performed on different sub-populations. Phylogroup B (strain RC) had negative Tajima's D = −0.161. The exclusion of isolate P33 from phylogroup B generated a positive Tajima's D = 0.274 value. Uganda isolates (strain EA) and Kenya isolates (strain EA) had both positive D* and F* value and were all significant. EA-I had positive and significant values D* = 1.680, *P < 0.02* and F* = 1.666, *P < 0.05.* O-II cluster had positive Tajima and Fu & Li's values. However, only values D* = 1.729, *P < 0.02* were considered significant for O-II cluster.

**Table 3:** Neutrality test based on variation within polyprotein encoding nucleotide sequence.

	n	Tajima's D statistic	Fu & Li's D* statistic	Fu & Li's F* statistic
All isolates	29	0.223, *P > 0.10*	2.389, *P < 0.02*	1.827 *P < 0.05*
EA-I	5	0.856, *P > 0.10*	1.680, ***P < 0.02***	1.666, ***P < 0.05***
EA-III	6	0.229, *P > 0.10*	1.701, ***P < 0.02***	1.497, *P* = *0.10*
O-II	7	0.248, *P > 0.10*	1.729, ***P < 0.02***	1.518, *P* = *0.1*
Phylogroup B	6	−0.161, *P > 0.10*	1.696, ***P < 0.02***	1.376, *P > 0.10*
Uganda isolates (Strain EA)	10	0.488, *P > 0.1*	1.83667, ***P < 0.02***	1.666***, P < 0.05***
Kenya Isolates (Strain EA)	3	1.339, *P > 0.10*	1.770***, P < 0.02***	1.848, ***P < 0.02***
O-II East Africa origin (K16 and K31)	2	2.333, ***P < 0.01***	2.41273, ***P < 0.02***	1.482, *P* = *0.1*
O-II (non-East Africa origin)	5	0.691, *P > 0.10*	2.41273, ***P < 0.02***	1.482, *P* = *0.10*
Phylogroup B excluding P33	5	0.274, *P > 0.10*	**1.667, *P < 0.02***	1.490, *P* = *0.1*

Bold indicate significance neutrality tests at p = 0.05, number of isolates (n).

### 3.7 Selection pressure within SPFMV

More codons were under positive selection in *P1* gene compared to other genes as predicted by both MEME and IFEL. MEME predicted 25 codons and IFEL predicted 17 codons within *P1* gene to be positively selected. Both IFEL and MEME agreed that 10 codons were under positive selection within gene *P1* (Table 4). The following codon sites were predicted by at least two models to be under positive selection: codon site 215 in gene *P3*, 57 in gene *CI*, 27 in gene *6K2*, 196 in gene *NIa-Pro*, 49 in gene *NIb*, and 2 and 22 in gene *CP* (Table 4).

**Table 4:** Codon sites predicted under positive selection.

Gene	IFEL	REL	MEME
P1	**8,** 35, **82**, **248**, 256, **263**, 267, **270**, **271**, 272, **291**, 294, **495**, **561**, 578, **668**, 669	No positive selection site	**8,** 69, **82**, 89, 94, 117, **248**, **263**, **270**, **271**, **291**, 339, 346, 352, 362, 367, 400, 423, 430, 473, **495**, 533, **561**, **668**, 703
HC-Pro	353	No positive selected site	3
P3	131, **215**	334	81, 103, 108, 119, 152, 155, 164, 191, 192, **215**, 278, 335
6K1	No positive selected sites	No positive selected site	No positive selected sites
CI	**57**, 120	No positive selected site	**57**, 73, 105, 422
6K2	**27**	No positive selected sites	**27**
NIa-VPg	No positive selection	98, 118	177
NIa-Pro	No positive selected site	**196**	**196**, 198
NIB	**49**, 470, 515	**49**	336, 368, 400,468, 488
CP	**2**, 71	**2**, 4,13, 21, **22**, 65	**22**, 259, 276

Bold indicate sites predicted by two models to be under positive selection.

### 3.8 Recombination events within genome of SPFMV

Out of 16 isolates from the study, eight (K14, P35, U10, U33, U40, K3, U25, U32) showed evidence of recombination supported by strong p-value by at least four of the detection methods (Table 5). Similarly, eight of the isolates showed no evidence of recombination occurring within their genome. Many of the recombination occurrences involved isolates of phylogroup A. Isolates K16, K31, AB465608, AB439206, KF386013, KF386014, MF572056 all belong to O-II subgroup and were parents of most recombinants detected in this study (Table 5). AB465608 was predicted as the parent of several recombinant isolates including; K14, U10, P35, K3 AB439206, FJ155666, KP729265.1. Isolate K14 was predicted to have derived nucleotide fragments of 1783–10595 from major parent (AB465608). Generally, recombination in phylogroup B (strain RC) were very rare and no isolates from East Africa had recombination events with phylogroup B. Six of the recombination starts point were found within *P1* gene, two were found within *HC-Pro* gene, two within *NIa-Pro* gene, and two within *NIa-VPg*. Recombination break points for recombinant isolate U33 was located within the junction that separate *NIa-Pro* and *NIa-VPg* genes (nucleotide position = 7316). Ten of the recombination end points were within *CP* gene.

**Table 5:** Predicted recombinant isolates, recombination breakpoints and parental-like isolate.

Breakpoint in Recombinant						Detection Methods				
Begin	End	Recombinant	Minor Parent	Major Parent	RDP	G	B	M	C	S
1013	10415	AB439206	D86371	AB465608	5.99E-70	3.81E-91	5.73E-86	1.87E-24	8.08E-25	8.44E-27
7483	10613	FJ155666	P35	AB465608	4.43E-74	NS	9.28E-85	5.73E-36	2.48E-38	1.01E-37
6656	10471	K14	U25	AB465608	7.54E-65	1.23E-57	1.03E-70	5.77E-39	6.27E-40	6.16E-39
7596	10276	KP729265.1	P35	AB465608	3.51E-66	NS	1.72E-70	1.54E-35	1.00E-35	7.10E-34
22	3305	P35	AB465608	Unknown (P33)	1.49E-69	1.01E-69	2.73E-77	1.08E-32	4.08E-33	1.55E-45
8410	9994	U10	P35	AB465608	4.09E-68	3.31E-45	9.02E-64	9.49E-22	1.09E-22	6.24E-25
7316	10263	U33	P35	AB439206	1.81E-51	NS	8.05E-61	2.78E-35	4.80E-36	1.58E-35
1089	10476	U40	Unknown (U10)	K3	6.98E-18	8.91E-39	6.43E-50	4.92E-19	1.20E-10	4.31E-21
6825	7470	K3	AB465608	U26	1.39E-45	1.57E-39	1.69E-45	1.71E-16	4.07E-17	4.40E-13
3316	10011	K3	AB465608	Unknown (P33)	3.85E-50	2.39E-61	3.22E-69	1.69E-33	8.54E-26	7.12E-71
6788	7312	U25	MF572056	P35	1.50E-30	5.54E-12	4.26E-29	5.53E-12	8.77E-12	2.23E-08
842	10509	U32	Unknown (MF572055.1)	U44	4.72E-27	4.54E-17	4.69E-24	1.06E-16	1.21E-12	4.03E-12
9195	9523	MF572056	KF386014	KF386013	1.81E-25	4.41E-22	NS	4.72E-08	1.72E-08	1.08E-07
3384	3474	U33	U23	K16	5.09E-07	2.76E-05	1.83E-07	2.87E-02	NS	5.08E-53
1014	1255	MF572056	MF572047	K31	1.31E-08	NS	2.04E-07	1.84E-02	4.81E-03	1.15E-03
843	1279	FJ155666	Unknown (K16)	U32	2.86E-06	6.05E-05	8.51E-07	4.67E-06	1.21E-04	4.07E-14
6592	8409	U40	U7	K31	8.53E-03	6.01E-07	6.07E-06	4.23E-04	1.26E-05	4.98E-12
6638	10421	KP115608	P35	KF386013	3.30E-06	NS	4.38E-07	3.15E-22	4.10E-21	2.53E-49

Non-significant (NS), GENECONV (G), BootScan (B), MaxChi (M), Chimaera (C), SiScan (S).

### 3.9 Characterization of nucleotide repeat

Phylogroup A had a satellite repeat (CAACAG) occurring three times at nucleotide positions 94–111 within the *P1* gene. At the same position phylogroup B (strain C) had a satellite repeat (CAG) that occurred six times. Other repeat regions that only occurred within *P1* gene of phylogroup B include; CTGCT (positions 224–233), AACC (953–960), and AGCA (2093–2100). A repeat with three iterations CGTG (1908–1918) and CGA (1941–1950) and repeat with four iteration GT (2390–2397) occurred only in phylogroup B. Mononucleotide repeats of five iterations varied in all the isolates of SPFMV at different locations within the genome. Isolate D86371 had 16 pentanucleotide (A_5_) repeats, followed by isolate P33 with 15 repeats. U40 had the least pentanucleotide repeat A (Table 6). Isolates U33, U26 and K5 had the highest occurrences of pentanucleotide T (T_5_) repeats. Pentanucleotide C (C_5_) repeats were least common, with the highest occurrence in isolates K14, K31 and K38. Isolate U32 and reference sequence KF386014 lacked pentanucleotide C repeat within the genome.

**Table 6:** Variation in the number of mononucleotide repeats among isolates of SPFMV.

Isolates	AAAAA	CCCCC	GGGGG	TTTTT
KF386014	14	0	5	6
D86371	16	1	4	7
P33	15	1	2	4
AB465608	10	2	5	8
KF386013	12	2	5	9
K31	10	3	6	9
K16	11	2	6	6
K14	10	3	6	9
K3	12	1	3	9
K5	11	2	4	11
P35	11	1	4	7
U10	12	2	5	10
U23	12	2	4	9
U25	13	2	6	9
U26	11	1	3	11
U32	11	0	4	8
U33	11	2	6	11
U40	8	2	5	9
U44	9	1	7	9
U7	13	2	5	10

## 4 Discussion

This study presents a genome characterisation of SPFMV isolated from East African countries − Kenya, Rwanda, and Uganda as a contribution to the body of knowledge on the genetic diversity and evolution of an important sweet potato virus. A phylogenetic tree of polyproteins separated the isolates into two main phylogroups: A and B. Our groupings are similar to those obtained by other researchers [9]. Phylogroup A had isolates of strain O and strain EA according to earlier classification [7]. Subgroup O-II (Strain O) appears to be less diverse as it formed only one sub-cluster compared to subgroups EA (strain EA) which had three different sub-clusters (EA-I, EA-III, EA-IV) that appear to be geographically shaped. The EA-III subgroup had isolates from Kenya, Uganda and Rwanda. EA-I were variants restricted to Uganda and EA-IV were strain EA isolated outside East Africa. The phylogenetic tree shows that O-II (strain O) and EA-I, EA-III, and EA-IV (strain EA) had a monophyletic origin and are distantly related with phylogroup B (Strain RC).

Some of the unique features shared by isolate P33 and phylogroup B include; deletion of 75 nucleotides in *P1 gene*, sequence repeat (CAG)6 within the *P1* gene, KRTV and LEPV amino acid motifs in *P1 gene*. Isolate P33 had high nucleotide identity with phylogroup B compared to nucleotide identity with phylogroup A. Isolate P33 cluster with phylogroup B in phylogenetic tree. The above features support that isolate P33 belong to phylogroup B (strain RC). Similarly, isolates K16 and K31 had high nucleotide identity and cluster with O-II subgroup which indicate they belong to subgroup O-II of phylogroup A. Out of the 16 isolates, two belonged to O-II (strain O), one belonged to phylogroup B and the rest were in the EA subgroup (East Africa strain). Our findings support earlier studies that reported a low occurrence of strain O within East Africa [11]. We also report the occurrence of phylogroup B (strain RC) in East Africa for the first time, albeit with a limited distribution. Some sweet potato cultivars had developed resistance to SPFMV and SPCSV infection that results to SPVD [26,27]. Such resistance was mainly developed toward the native SPFMV phylogroup A (specifically to strain EA) in east Africa. Thus, the new phylogroup B may present disease challenge to sweet potato varieties as resistance mechanisms may be specific and developed over time as result of host-pathogen interaction and co-evolution. Co-infection involving both phylogroups and their variants together with SPCSV may be possible as the prevalence of the phylogroup B increase. The scenario can be investigated to quantify the associated yield losses from the interaction.

The comparison of nucleotide diversity from our study indicates relatively high nucleotide diversity among SPFMV isolates from Uganda and Kenya, in contrast to the diversity of isolates from other geographical regions. Similar findings of high genetic diversity among SPFMV isolates in East Africa have been reported [3,11] and led to the conclusion that East Africa was hotspot for diversification of major plant viruses. O-II (strain O) of East Africa origin (K16 and K31) shows low nucleotide diversity; this is similar to nucleotide diversity of O-II (strain O) of non-East African origin, indicating genetic stability within O-II subgroup in contrast to that in EA subgroups (strain EA).

The limited gene flow and high genetic differentiation among the different sub-population of SPFMV noted in this study indicate restricted long-distance spread of SPFMV from other continents into East Africa. Restricted gene flow of SPFMV into east Africa from geographical region outside east Africa is associated with low risk of introduction of alien strain/variants of SPFMV in to east Africa. The alien variants may breakdown the resistance traits of local sweet potato varieties acquired against local variants of SPFMV hence posing threat to production. Cross-continental spread of plant pathogen usually occur unknowingly through the trade of infected plant materials. The only evidence of gene flow was between isolates from Kenya and Uganda: this might be expected as these two countries border one another. It is likely that farmers located in the border areas of both countries share vines which could subsequently be distributed to other sweet potato farmers in different parts of the two countries. Gene flow between Uganda and Kenya is not limited to SPFMV as we show in this study; it has been reported also for other plant viruses [28]. Isolates of subgroup O-II (K16 and K31) of East Africa origin show high genetic differentiation with O-II isolate of non-East Africa, suggesting that the separation within this group might have taken place long ago and that the mechanism that introduced them to east Africa is currently not active.

Recombination events were common between O-II and EA population of phylogroup A compared with those in phylogroup B (strain RC). The phylogeny tree from this study and that of [9] indicate that O-II and EA subgroups belong to the same phylogroup. Nucleotide identity further indicates that both O-II and EA groups are closely related to each other but distantly related to phylogroup B. Recombination events between distantly related isolates produce progeny with a decreased ability to survive − as shown in cucumber mosaic virus [29]. That could explain the observed occurrence of more recombination events between O-II and EA groups of phylogroup A as observed in our study. Evidence of recombination events occurring between O-II and EA group was reported between isolate FJ155666 (Piu3) from Peru and 10-O [10]. Similarly, our study indicates that isolate FJ155666 (Piu3) is recombinant with two major parents; first was isolate AB465608 of O-II and isolate U32 of EA-I. Reference isolate AB465608 was the parent for most recombinant isolates of EA group. The recombination events among O-II (non-East Africa origin) and EA group (both Africa and non-Africa origin) provide evidence that they shared the same geographical region possibility outside east Africa as occurrence of both O-II and phylogroup B is limited within east Africa. It provides evidence that can be used to resolve controversies on the origin of EA subgroup of SPFMV [3,9,11]. EA groups had a high nucleotide diversity and recombination just like phylogroup B had low nucleotide diversity and recombination. The correlation between nucleotide diversity and recombination indicates that the latter is a factor contributing to genetic diversity among EA isolates.

Many sites within *CP, NIB, NIa-Pro, NIa-Vpg, P3, CI* genes were found to be under negative selection rather than positive selection, indicating that the genes were constrained by major changes within the proteins. Gene *6K1* had no site under positive selection, while gene *6K2* and gene *CI* had only one site predicted under diversification. Our study confirmed seven of nine codons that were first reported to be under positive selection within *CP* gene [11]. However, we further identified another two codons (259 and 276) toward C-terminal of *CP* gene to be under positive selection. Codon 2 and 22 within the coat protein were predicted by two models to be under positive selection within the *CP* gene which increase the confidence that they have undergone positive selection. Some of the codons under positive selection in *CP gene* closely border DAG motif which aid aphid transmission of the virus as previously reported [11]. Similarly, positive selected sites 336, 400, 468 border motif SG(X)_3_T(X)_3_NT(X)_30_GDD [30] which play role in RNA-dependent RNA polymerase (RdRp) in potyvirus *NIb* gene. Positive selected site 353 in *HC-Pro* gene occurred within motif GYCY(X)_71_H with proteolytic activity. Similarly, codon site 105 was within motif VLLLEPTRPL in CI gene [30]. The positive selection of codons bordering or within motifs at different genes is an indication of an adaptive evolution within the virus protein to increase their ability to survive within the host.

Short sequence repeats provide a useful tool for understanding genetic diversity among viruses [31]. Our study present short sequence repeats that have evolved differently between phylogroup A and phylogroup B; these could be useful in SPFMV genotyping. Sequence repeat have been used in genotyping different viruses including herpes viruses [32]. The sequence repeats at nucleotide position 94–111 can provide tool for genotyping phylogroups of SPFMV and monitor co-infection of sweet potato plant by both phylogroup A and B in addition to restriction fragment length polymorphism (RFLP) used to determine inter strain/variants co-infection [11]. As well, it can be useful in monitoring the occurrence and distribution of phylo-group B within sweet potato production region in east Africa. SPFMV isolates showed variation in the number of mononucleotide repeats for different isolates; this variation can be used as a measure of the extent of genetic diversity among the isolates. Mononucleotide A and T repeats were common with high frequency within the SPFMV genome, compared with G and C repeats; similar findings have been reported for other viruses [33]. High occurrences of A_5_ and T_5_ repeats compared with repeats of G_5_ and C_5_ is due to A and T forming a less strong bond compared to G and C [34]. In the genus *Potyvirus*, repeat of mononucleotide A was associated with a polymerase slippage site that is known to generate a new reading frame for the *PIPO* and *PISPO* coding regions with consensus G2A6 [1].

## 5 Conclusion

This work provides an analysis of the diversity of SPFMV and its evolution as an agriculturally important pathogen. Our phylogenetic analysis clustered the isolates into two main phylogroups, namely A and B. The new phylogroup B may present new disease challenge to sweet potato varieties in east Africa. Nucleotide diversity of O-II group isolated from east Africa and those isolated elsewhere was nearly the same and lower than that of EA group particularly those isolated from Kenya and Uganda. The east Africa isolate of phylogroup B is distantly related to phylogroup B isolated outside east Africa as shown by low nucleotide identity and high number of nucleotide differences. Much as the O-II group had low genetic diversity, the genetic differentiation between O-II of east Africa and those isolated outside east Africa was high. The limited gene flow and high genetic differentiation between the isolates from east Africa and those isolated elsewhere indicate restricted movement of the virus from different geographical areas into east Africa. Recombination events were found to be common among isolates of phylogroup A compared with phylogroup B. The recombination contributes to the diversity among SPFMV isolates of EA group from East Africa. Some of the sites under positive selection where bordering or occurred within motifs indicating an ongoing evolutionary adaption within SPFMV. Nucleotide repeat region within the *P1* gene provide basic difference between phylo group A and B which can be potential molecular marker to monitor the distribution of phylogroup B within east Africa.

## Availability of data and materials

The genome sequences resulting from this study were deposited into the GenBank database under the accession numbers from MH763675–MH763686.

## CRediT authorship contribution statement

**Godfrey Wokorach:** Conceptualization, Funding acquisition, Formal analysis, Writing - original draft. **Geoffrey Otim:** Conceptualization, Formal analysis, Writing - review & editing. **Joyce Njuguna:** Formal analysis, Writing - review & editing. **Hilary Edema:** Formal analysis, Writing - review & editing. **Vincent Njung'e:** Conceptualization, Writing - review & editing. **Eunice M. Machuka:** Conceptualization, Writing - review & editing. **Nasser Yao:** Conceptualization, Writing - review & editing. **Francesca Stomeo:** Conceptualization, Funding acquisition, Writing - review & editing. **Richard Echodu:** Conceptualization, Funding acquisition, Writing - review & editing.

## Declaration of competing interest

None.
