# Supplementary material for: Genomic analysis of Sweet potato feathery mottle virus from East Africa
Source: Physiol Mol Plant Pathol. 2020 Apr;110:101473. doi: 10.1016/j.pmpp.2020.101473 (PMC7233136; doi:10.1016/j.pmpp.2020.101473)
Supplement: Multimedia component 2 [file mmc2.doc]

Table S 1 Genome characteristics of the SPFMV isolates used in this study

| Sample ID | GenBank accession | Country | Local area | Genome  length in nucleotides (nt) | Polyprotein  length (nt) | Polyprotein  length (AA) | Nucleotide composition of the genome  (%) | | | | 5′ UTR | 3′ UTR |
| --- | --- | --- | --- | --- | --- | --- | --- | --- | --- | --- | --- | --- |
|  |  |  |  |  |  |  | A | C | G | T |  |  |
| U32 | MH763678 | Uganda | Iganga | 10879 | 10557 | 3518 | 32.5 | 18.8 | 23.0 | 25.7 | 102 | 220 |
| U44 | MH763690 | Uganda | Lira | 10868 | 10557 | 3518 | 32.5 | 18.8 | 23.1 | 25.5 | 90 | 221 |
| U40 | MH763679 | Uganda | Mukono | 10885 | 10557 | 3518 | 32.6 | 18.7 | 23.0 | 25.7 | 107 | 221 |
| U10 | MH763676 | Uganda | Masaka | 10880 | 10557 | 3518 | 32.5 | 18.6 | 23.1 | 25.7 | 104 | 219 |
| K31 | MH763675 | Kenya | Kakamega | 10887 | 10557 | 3518 | 32.7 | 18.8 | 22.9 | 25.6 | 109 | 221 |
| K16 | MH763689 | Kenya | Kirinya | 10891 | 10557 | 3518 | 32.6 | 18.8 | 23.1 | 25.5 | 116 | 218 |
| K14 | MH763680 | Kenya | Homabay | 10882 | 10557 | 3518 | 32.4 | 18.6 | 23.2 | 25.7 | 107 | 218 |
| U7 | MH763677 | Uganda | Mukono | 10868 | 10557 | 3518 | 32.7 | 18.8 | 23.1 | 25.5 | 104 | 207 |
| U33 | MH763681 | Uganda | Iganga | 10861 | 10557 | 3518 | 32.6 | 18.6 | 23.2 | 25.7 | 104 | 200 |
| K5 | MH763682 | Kenya | Nakuru | 10877 | 10557 | 3518 | 32.7 | 18.7 | 22.9 | 25.7 | 99 | 221 |
| U25 | MH763683 | Uganda | Lira | 10890 | 10557 | 3518 | 32.8 | 18.8 | 22.9 | 25.5 | 112 | 221 |
| U23 | MH763684 | Uganda | Arura | 10877 | 10557 | 3518 | 32.8 | 18.8 | 22.8 | 25.5 | 100 | 220 |
| K3 | MH763685 | Kenya | Nakuru | 10879 | 10557 | 3518 | 32.8 | 18.9 | 22.8 | 25.5 | 104 | 218 |
| P35 | MH763686 | Rwanda | Nyamagabe | 10868 | 10557 | 3518 | 32.7 | 19.0 | 22.9 | 25.4 | 104 | 207 |
| U26 | MH763687 | Uganda | Iganga | 10882 | 10557 | 3518 | 32.7 | 18.9 | 22.9 | 25.5 | 108 | 217 |
| P33 | MH763688 | Rwanda | Ngororero | 10803 | 10482 | 3493 | 32.2 | 18.8 | 23.4 | 25.5 | 100 | 221 |
